# L’arbre qui cachait la forêt: aspergillose sur carcinome bronchique (à propos de deux cas)

**DOI:** 10.11604/pamj.2017.28.302.11665

**Published:** 2017-12-08

**Authors:** Fatima Zahra Mrabet, Mouna Soualhi, Jihane Achrane, Yassir Sabri, Sanaa Hammi, Karima Marc, Jouda Benamor, Rachida Zahraoui, Jamal Eddine Bourkadi

**Affiliations:** 1 Service de Pneumologie, Hôpital Moulay Youssef, CHU de Rabat, Akkari, Maroc; 2 Laboratoire de Parasitologie et de Mycologie de l’Hôpital Ibn Sina, CHU Rabat, Maroc

**Keywords:** Aspergillome, endobronchique, intracavitaire, néoplasie bronchique, Aspergilloma, endobronchial, intracavitary, bronchial neoplasia

## Abstract

L’aspergillome endobronchique et pulmonaire intracavitaire peut mimer à une néoplasie bronchique cliniquement et radiologiquement, ainsi une recherche systématique d'une association est impérative. Une association confirmée change complètement le pronostic ainsi que la conduite thérapeutique. Nous présenterons les observations de deux patients présentant deux formes différentes d'aspergillome pulmonaire mais renfermant en leur sein un carcinome bronchique.

## Abstract (en)

Endobronchial aspergilloma and intracavitary pulmonary aspergilloma may clinically and radiologically mimic a bronchial neoplasia, hence the importance of systematically searching for an association. A confirmed association completely changes the prognosis as well as the therapeutic approach. We here report two cases with two different forms of pulmonary aspergilloma associated with bronchial carcinoma.

## Introduction

L’aspergillose est une mycose due à un champignon filamenteux. Il en existe 300 espèces dont une dizaine seulement sont pathogènes pour l’homme. L’espèce fumigatus est responsable des atteintes respiratoires [1]. L’aspergillome endobrochique est une forme rare de l’aspergillose pulmonaire, alors qu’une greffe aspergillaire pulmonaire est une situation assez fréquente favorisée par des terrains particuliers (présence préalable d’une cavité pulmonaire) même chez le sujet immunocompétent [2,3]. On rapporte l’observation de deux patients ayant deux présentations différentes de l’aspergillose pulmonaire associée à un carcinome bronchique. L’intérêt de notre présentation est d'attirer l'attention sur la probabilité d'association d’un néoplasie pulmonaire en cas d’aspergillose pulmonaire.

## Patient et observation

**Figure 1:**
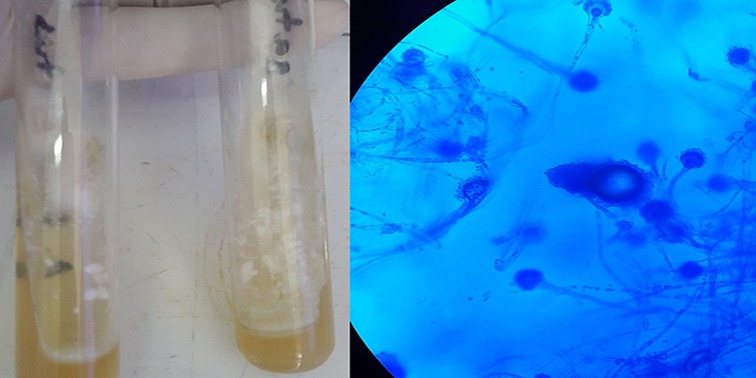
Aspect macroscopique des cultures d’aspergillus fumigatus (à droite) et examen direct à l’état frais de colonie d’aspergillus fumigatus avec bleu de lactophénol (à gauche)

La première observation décrit le cas d’un patient de 57 ans, tabagique chronique, sans antécédent pathologique particulier ayant rapporté deux mois avant sa consultation l’apparition des épisodes de détresse respiratoire spontanément résolutifs sans autre signe respiratoire ou extra-respiratoire associé, le tout évoluant dans un contexte d’altération de l’état général. L’examen clinique a objectivé un syndrome de condensation au niveau de tout l’hémi-thorax gauche. La radiographie thoracique de face a montré une opacité de tout l’hémi-champ thoracique gauche dense homogène avec attraction de la trachée, ascension de la coupole diaphragmatique gauche et pincement des espaces inter-costaux. Le complément scannographique a mis en évidence une atélectasie totale du poumon gauche avec individualisation en son sein d’une masse de 64mm. La bronchoscopie a objectivé la présence d’une formation noirâtre et friable obstruant complètement la bronche souche gauche. Les biopsies ont été en faveur d’un carcinome peu différencié non à petites cellules primitif bronchique, l’aspiration bronchique, a mis en évidence la présence de nombreux amas de filaments mycéliens dont l’étude mycologique a permis d’identifier l’espèce d’aspergillus fumigatus (Figure 1). Devant ces éléments cliniques, radiologiques, endoscopiques et les données des examens microscopique et immunohistochimique, le diagnostic d’aspergillome endobronchique associé à un carcinome bronchique a été retenu. Le patient a été mis sous itraconazole à la dose de 400 mg par jour avant de démarrer toute thérapeutique anti-cancéreuse. Une semaine après, l’évolution fut marquée par l’installation d’une détresse respiratoire entraînant le décès du patient dans un tableau d’asphyxie.

**Figure 2:**
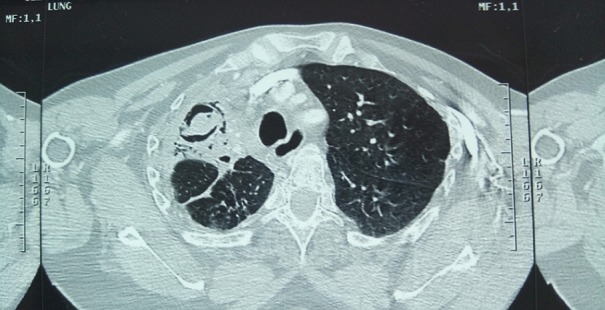
Image scannographique montrant un aspect en grelot

**Figure 3:**
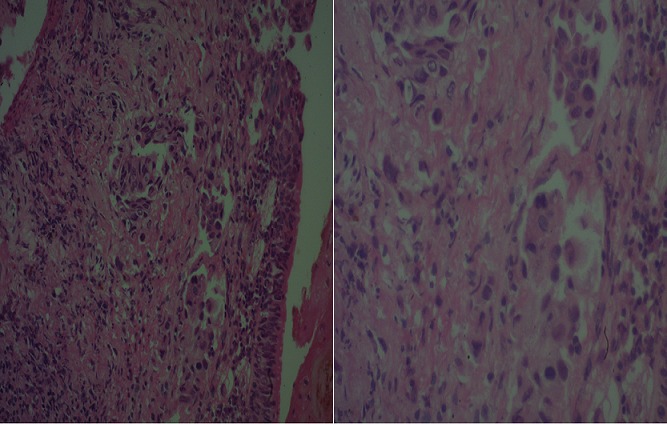
Aspect histologique de la prolifération carcinomateuse (HE G x 10) à droite et (HE G x 40) à gauche

La deuxième observation rapporte le cas d’un patient âgé de 55 ans, sans habitudes toxiques et sans antécédents notables, présentant depuis 6 mois une toux, des épisodes récurrentes d’hémoptysie de faible abondance et une dyspnée stade III de la mMRC, évoluant dans un contexte d’altération de l’état général. L’examen clinique a retrouvé un syndrome de condensation pulmonaire droit avec à la radiographie thoracique une opacité pulmonaire apicale droite surmontée d’un croissant gazeux. Le scanner thoracique a montré la présence d’une cavité pulmonaire apicale droite renfermant une image en grelot (Figure 2). Une tuberculose pulmonaire a été éliminée après des recherches de BK dans les expectorations qui sont revenues négatives, aussi le mycobarterium tuberculosis n’a pas été détecté par GenExpert. Le diagnostic d’aspergillome intracavitaire a été retenu devant la présence de l’image en grelot identifiée au scanner ainsi qu’une sérologie aspergillaire qui est revenue très positive. La bronchoscopie a objectivé une coulée tumorale descendante jusqu’à la lobaire supérieur droite qui est complétement sténosée par un bourgeon. L’étude histologique de fragment de biopsie du bourgeon a mis en évidence une prolifération carcinomateuse faite de massifs et de travées bordées de cellules polyclonales aux noyaux augmentés de taille nucléolés au sein d’un cytoplasme éosinophile abondant avec focalement des signes de différenciation malpigienne à type de pont d’union et de dyskératose et dont le complément immunohistochimique a été en faveur d’un carcinome non à petites cellules sans spécificité (marquage positif de TTF1 et P63) (Figure 3).

Le patient a été mis également sousitraconazole à la dose de 400 mg par jour avant de démarrer un traitement anti-cancéreuse. L’évolution a été marquée par le décès du malade par une complication infectieuse deux semaines plutard.

## Discussion

Les présentations habituelles de l’aspergillose pulmonaire sont l’aspergillose bronchopulmonaire allergique (ABPA), l’aspergillome, l’aspergillose nécrosante chronique et l’aspergillose invasive [4]. L’aspergillome se développe sous forme de colonisation par un conglomérat de filaments, de mucus et de débris cellulaires au niveau: Des voies aériennes (généralement pathologiques) ou des cavités pulmonaires pré existantes [1].

Les manifestations cliniques de l’aspergillome, rapportées dans les revues de la littérature, sont faites essentiellement de toux, dyspnée et hémoptysie [5-7]. Il n'y a aucun cas rapporté dans la littérature d'une détresse respiratoire fluctuante comme le cas de notre première observation. L’atteinte pulmonaire est habituellement décrite au niveau du lobe supérieur [8], ce qui concorde avec la présentation clinique et para-clinique de nos deux patients.

Le diagnostic de l’aspergillome endobrochique est presque toujours confirmé par un examen microscopique [9]. Celui de l’aspergillome intra cavitaire est posé généralement devant la présence d’une image en grelot sur le scanner (qui n’est pas toujours facile à identifier) avec une sérologie aspergillaire qui est quasiment toujours positive [10].

A l’heure actuelle, il n’existe aucune preuve de l’efficacité de traitement antifongique ni par voie systémique ni par instillation endobrochique ou intracavitaire. L’abstention thérapeutique reste la règle dans le cadre d’aspergillome endocavitaire notamment chez le patient asymptomatique. Cependant la chirurgie représente le seul traitement curatif et est réservée aux cas d’hémoptysie massive chez des patients dont la fonction respiratoire est adéquate [10]. En cas d’association d’aspergillose avec un carcinome bronchique, la prise en charge est complètement différente et nécessite la prise par considération de nombreux critères notamment le type histologique de la tumeur, le bilan d’extension tumoral, l’opérabilité (de la tumeur et du malade).

Le pronostic de l’aspergillome est surtout lié à la maladie pulmonaire sous-jacente notamment un néoplasie associée pouvant le rendre péjoratif surtout en absence de recherche systématique et soigneuse.

## Conclusion

L'aspergillome peut mimer un carcinome endobronchique cliniquement et radiologiquement. Ainsi un carcinome bronchique peut se cacher sous une couverture de champignon, de fibrine et de débris tissulaires. La recherche systématique d’une association néoplasique concomitante est impérative devant toute forme d’aspergillome pulmonaire en raison du chevauchement clinique et radiologique de ces deux diagnostics. Une identification précoce d’un carcinome bronchique a un intérêt dans la prise en charge ainsi que le pronostic.

## Conflits d’intérêts

Les auteurs ne déclarent aucun conflit d’intérêts.
